# Ultrasound combined with DSA-guided pulsed radiofrequency for perineal herpes zoster pain management: clinical outcomes and complications

**DOI:** 10.3389/fmed.2024.1442199

**Published:** 2024-11-11

**Authors:** Qing-Peng Dong, Shao-Jun Li

**Affiliations:** ^1^Department of Pain Management, Geriatric Hospital Affiliated with Wuhan University of Science and Technology, Wuhan, Hubei, China; ^2^Department of Pain Management, Wuhan No.1 Hospital, Wuhan, Hubei, China

**Keywords:** ultrasound guidance, pulsed radiofrequency, perineal herpes zoster, pain, complications

## Abstract

**Background:**

Herpes zoster (HZ) in the perineal area is a rare disease. There are limited treatment options for this disease. This study aimed to assess the efficacy of ultrasound combined with digital subtraction angiography (DSA)-guided pulsed radiofrequency (PRF) for perineal herpes zoster-related pain.

**Methods:**

Two hundred and twelve patients with perineal HZ were enrolled at the pain department. From January 1, 2018 to December 30, 2019, patients with HZ in the perineal area were treated with PRF under DSA guidance, and from January 1, 2020 to October 30, 2023, patients with HZ in the perineal area were treated with PRF under ultrasound combined with DSA guidance. The included patients were divided into two groups: DSA group and ultrasound + DSA group. The visual analog scale (VAS), central sensitization inventory (CSI), sleep quality scores (SQS), the 36-Item Short Form Health Survey questionnaire (SF-36) at baseline and after the PRF treatment were analyzed to evaluate clinical efficacy.

**Results:**

The mean VAS scores, CSI scores, SQS, and SF-36 scores were statistically significantly lower after treatment compared to baseline (*p* < 0.001). There was no significant change in VAS scores, CSI scores, SQS, and SF-36 scores between the DSA group and the ultrasound + DSA group (*p* > 0.05). The time it took from the start of the puncture to the successful puncture was significantly shorter in the ultrasound + DSA group compared to the DSA group (41.2 ± 21.2 vs. 48.1 ± 20.3, *p* = 0.035). The ultrasound + DSA group had a higher percentage of satisfaction with the procedure than the DSA group (90/99, 90.9% vs. 62/78, 79.5%, *p* = 0.030). A total of 27 postoperative complications occurred. The incidence of puncture site hematoma was significantly higher in the DSA group (10/78, 12.8%) than that in the ultrasound + DSA group (4/99, 4.0%) (*p* = 0.032).

**Conclusion:**

PRF can reduce pain from HZ in the perineal region, alleviate central sensitization, enhance sleep quality, and improve overall quality of life. When PRF is performed under ultrasound guidance combined with DSA, it shortens the puncture time and reduces the risk of hematoma formation at the puncture site, making it the recommended method for clinical use.

## Introduction

Herpes zoster (HZ) in the perineal area is primarily characterized by shingles in the perianal area, urethra, and surrounding tissues. In a small number of patients, it may also present with symptoms such as urinary frequency, urgency, retention, constipation, and even urinary and fecal incontinence. HZ in the perineal region is rare, only 5% of cases was reported in the sacral dermatomes (1). But it can lead to complications such as postherpetic neuralgia. This condition can have a significant impact on sleep quality and overall well-being, often causing psychological distress and mental health issues in affected individuals.

Medication is the main form of treatment for perineal HZ-related pain. These medications typically include antivirals (such as acyclovir, valacyclovir, and famciclovir), anti-neuralgic drugs (such as gabapentin and pregabalin), and tricyclic antidepressants (2). Nonsteroidal anti-inflammatory drugs (NSAIDs) and opioid analgesics may also be prescribed for patients experiencing severe pain (3). Herpes usually disappears gradually with medicine and pain treatment. However, some patients develop postherpetic neuralgia, in which the pain is difficult to control (4). Unfortunately, if medication does not cure the discomfort, there is no other effective treatment for perineal HZ-related pain.

Digital subtraction angiography (DSA) is increasingly being used in pain medicine, mainly for the diagnosis and treatment of various pain-related disorders. Previously, treatment in pain departments was mainly guided by computed tomography (CT). CT-guidance is the use of X-ray rotational scanning to obtain tomographic images of *in vivo* structures. It is usually used to examine the morphology and location of organs, tissues, or lesions (5–7). However, the imaging speed is relatively slow and is suitable for static structural observation, and the radiation dose may be high, especially in the case of multiple scans (8). The DSA technique uses a lower amount of contrast to obtain clear Imaging provides high quality images of blood vessels and nerves. DSA allows dynamic observation of nerve distribution, ensures accurate injection of drugs into the target area, reduces the impact on surrounding tissues, and improves the success rate of nerve block or pulsed radiofrequency treatments, thus improving pain management outcomes for patients (9). Therefore, DSA provides effective imaging support for pain management, which can help doctors locate the puncture target more accurately and perform the corresponding interventional therapy. With the advantages of high-resolution imaging, real-time observation capability and reduced risk of complications, it has become an important tool for pain physicians.

At present, chronic perineal pain has been effectively managed by ganglion impar block (10, 11). The ganglion impar or Walther is a solitary retroperitoneal organ located at the sacrococcygeal junction. It is created by the terminal merger of the two paravertebral sympathetic chains (12). Ghai et al. (13) reported that ultrasound-guided ganglion impar block was technically feasible and safe technique for chronic perineal pain. However, in another study, it was found that 43.6% of patients with chronic refractory pelvic and perineal pain had pain improvement after three repeated ganglion impar blocks. Repetitive ganglion blocks reduced pain intensity in the short term, but the medium-term effect was moderate and no long-term follow-up was performed (14). Recently, pulsed radiofrequency (PRF) has become an effective treatment for pain. Li et al. (15) reported that ultrasound-guided ganglion impar block combined with PRF was a safe and effective way to treat perineal pain. Lazzari et al. (16) concluded that PRF of the sphenopalatine ganglion can offer a safe, minimally invasive and effective treatment for medically refractory chronic cluster headache. Tak et al. (17) reported that intra-articular PRF therapy was a beneficial treatment tool for managing refractory chronic atlanto-occipital joint pain. A series of studies have shown PRF was an effective and safe treatment for herpes zoster-related pain (18–20). PRF is made up of a radiofrequency machine that creates high-frequency currents that are administered intermittently in pulses that typically last 20 milliseconds and have intervals of 480 milliseconds between intermittent and resting periods (21). This design limits the development of isolated high temperatures, hence avoiding nerve injury. PRF typically runs at frequencies ranging from 3 to 500 kHz, with maximum temperatures of no more than 42 degrees Celsius, which is insufficient to cause nerve injury or protein denaturation (22, 23).

Although PRF is an effective method for pain, there are no reports of PRF for HZ-related pain in the perineal area. The purpose of this article is to assess ultrasound combined with DSA-guided PRF for perineal HZ-related pain.

## Methods

### Patient selection

This was a retrospective observational study. Two hundred and twelve patients with perineal HZ were enrolled at the pain department of Geriatric Hospital Affiliated with Wuhan University of Science and Technology and Wuhan No.1 hospital between January 1, 2018 and October 30, 2023. All included patients were required to sign an informed consent form. This study received ethical approval from the Institutional Research Board of Geriatric Hospital Affiliated with Wuhan University of Science and Technology and Wuhan No.1 hospital. From January 1, 2018 to December 30, 2019, patients with herpes in the perineal area were treated with pulsed radiofrequency under digital subtraction angiography (DSA) guidance, and from January 1, 2020 to October 30, 2023, patients with herpes in the perineal area were treated with pulsed radiofrequency under ultrasound combined with DSA guidance. The included patients were divided into two groups: DSA group and ultrasound + DSA group.

**Figure 1:**
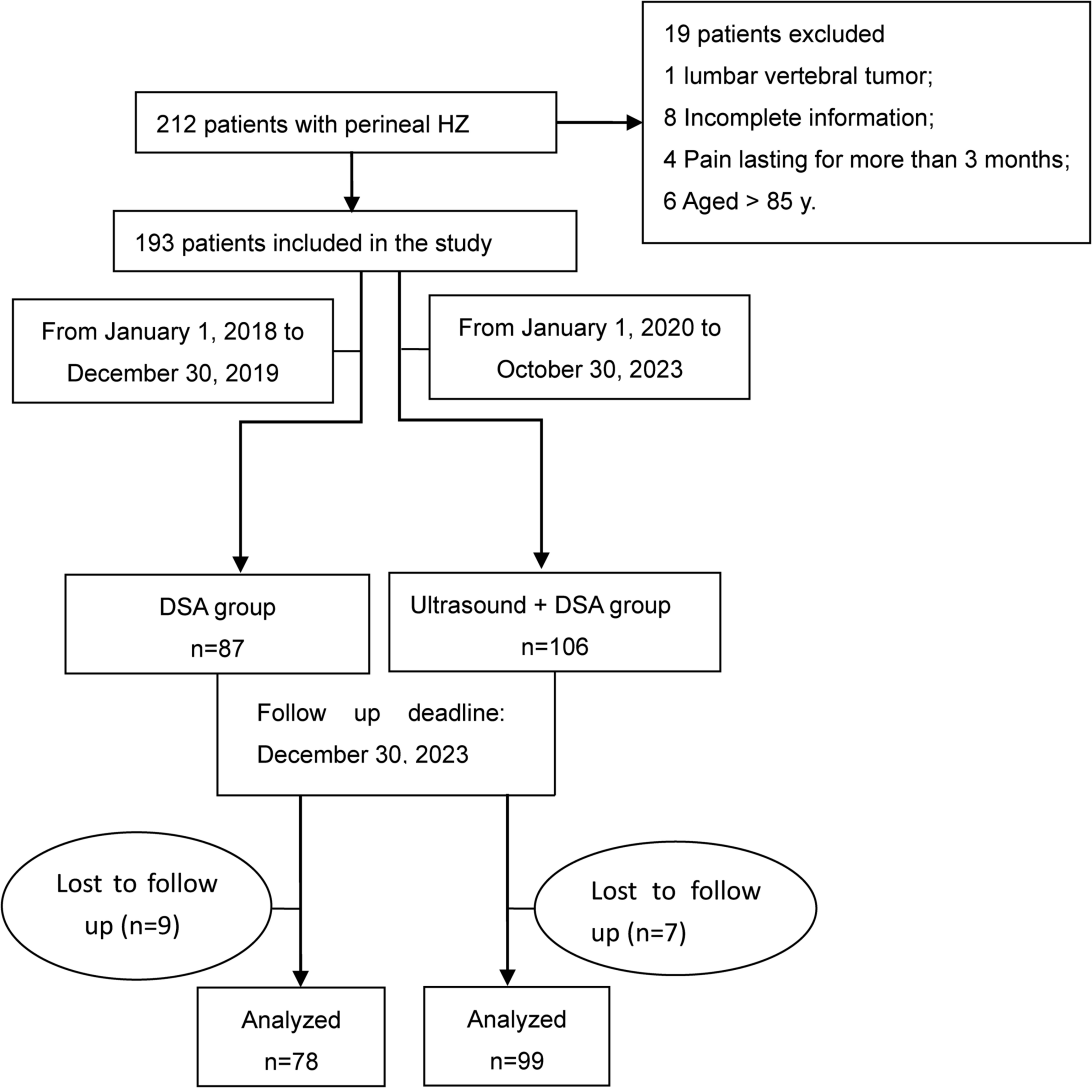
Patient inclusion flowchart. HZ, herpes zoster; DSA, digital subtraction angiography.

The inclusion criteria were physician-diagnosed perineal HZ; visual analog scale (VAS) score ≥ 4 before therapy; age > 18 years;

The exclusion criteria were inconsistent positioning between ultrasound and DSA; lumbosacral vertebral tumors; incomplete information; pain lasting longer than 3 months; age > 85 years; severe hepatic impairment or impaired renal function; blood coagulating dysfunction; systemic infection or puncture site infection (Figure 1).

### Measurements

#### DSA guided PRF process

**Figure 2:**
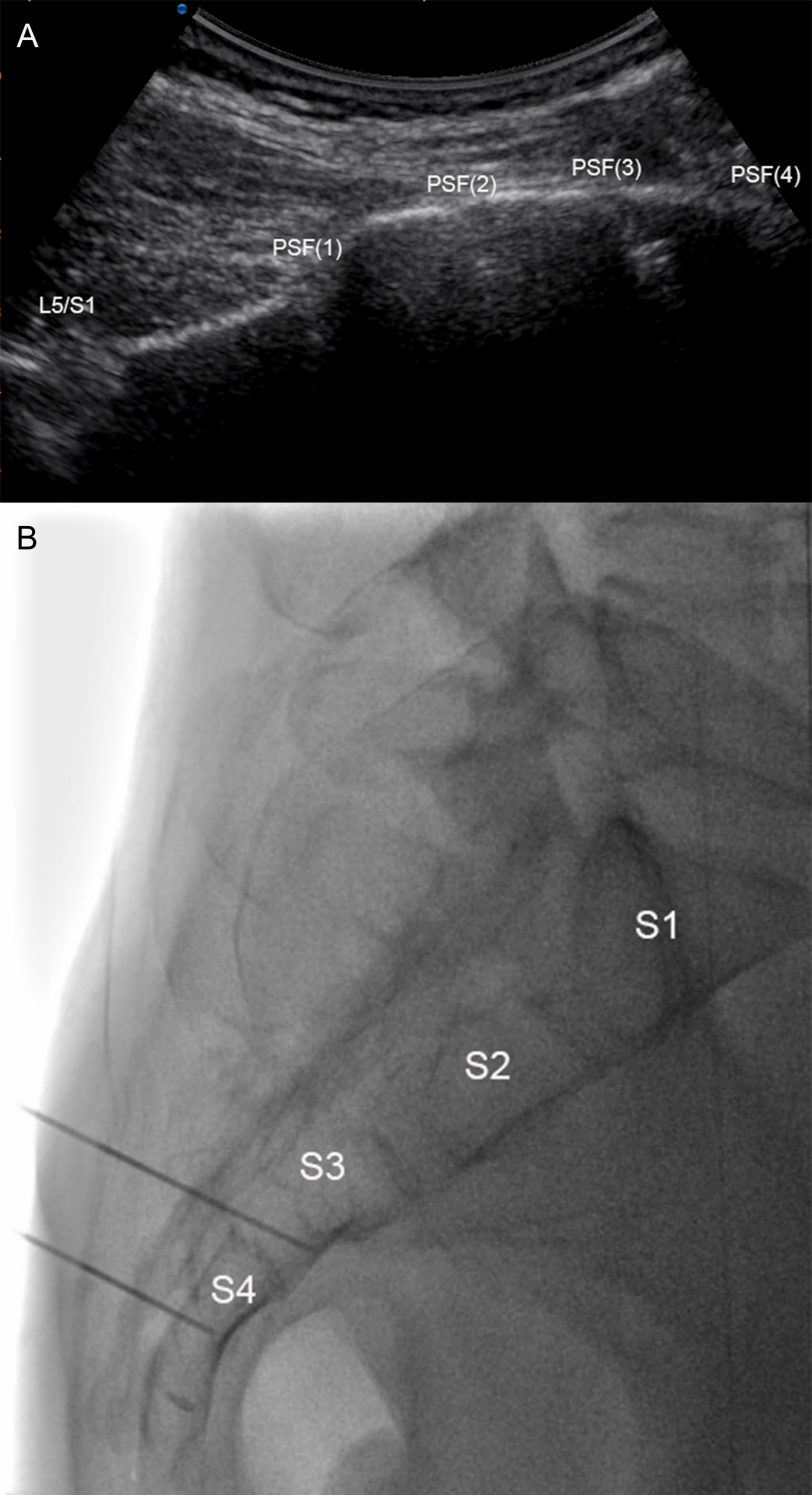
Ultrasound combined with DSA-guided pulsed radiofrequency for perineal herpes zoster. (A) Longitudinal ultrasound view of the posterior sacral foramen. (B) X-ray in the lateral position. The radiofrequency needle passed through the sacral foramen (S3, 4), with the tip of the needle located in front of the sacral foramen. PSF: posterior sacral foramina.

The patient was positioned prone with a pad supporting the pelvis, while being monitored with electrocardiograms. A disinfected and sterile sheet was placed, followed by administering 2 mL of 1% lidocaine for local skin anesthesia. Using DSA guided orthopantomogram, a posterior sacral foramen was identified and a 22-gauge radiofrequency needle was carefully passed from the posterior to the anterior sacral foramen. A lateral view was obtained to ensure the positioning of the radiofrequency needle tip at the anterior edge of the presacral foramen. It is important to ensure that the radiofrequency puncture needle does not extend beyond the anterior edge of the sacrum to avoid potential injury to pelvic organs (Figure 2B). The needle was carefully positioned to ensure that the tip was near the sacral nerve root location under the sensory stimulation testing (50 Hz, 0.5 V). Radiofrequency treatment began when the range of sensory stimulation closely matched the patient’s pain area. PRF was applied with 42°C, 2 Hz, 20 ms, 3 cycles (2 min each). Following the PRF treatment, a mixture of analgesic solution consisting of 1 mL of 2% lidocaine, 1 mL of betamethasone, and 3 mL of saline was injected around the sacral nerve.

#### Ultrasound-guided puncture process

The ultrasound probe is positioned in the para-sacral region for long-axis scanning to locate the L5/S1 joint and the sacrum. The first concave surface near the sacral surface of the L5/S1 joint is the S1 posterior sacral foramen, then followed by the S2, S3, and S4 posterior sacral foramina (Figure 2A). Target points were marked on the patient’s skin, and DSA-guided radiofrequency treatment was then carried out in accordance with the procedure outlined above.

#### Questionnaire

VAS is a method used to assess the intensity of pain. It involves using a 10 cm long measuring tape with a “0” and a “10” at each end. A score of 0 indicates no pain while a score of 10 represents the most severe and intolerable pain. The patient can mark their pain sensation on the scale, and the doctor can then assess the patient’s pain level based on this position. Central Sensitization Inventory (CSI) was used to assess whether a perineal HZ patient was centrally sensitized. The CSI is a reliable and pertinent evaluation tool that examines the symptoms of central sensitization through straightforward questions. A multi-and inter-disciplinary team specializing in assessing and treating patients with chronic pain conditions developed this questionnaire The CSI contains two sections, Parts A and B. Part A includes 25 items with a score range of 0–100, representing a variety of presenting symptoms commonly seen in central sensitivity syndrome (CSS). Higher scores indicate a greater level of symptom severity. Part B assesses if individuals have been diagnosed with certain CSS-related disorders by a physician, as well as associated conditions like anxiety and depression (24). Sleep quality scores (SQS) which contain five-item questionnaires were used to assess the sleep quality (25). The 36-Item Short Form Health Survey questionnaire (SF-36) was utilized to assess health-related quality of life (26). All patients included in the study were assessed before the treatment. Patients were assessed again 3 years after the treatment. The deadline for follow-up was December 30, 2023.

#### Outcome measures

The primary research purpose was the efficacy of PRF on reducing perineal HZ pain, based on the pain intensity, central sensitization intensity, sleep quality, and quality of life. The secondary research purpose was the effectiveness of reducing perineal HZ pain under the guidance of DSA-guided PRF alone or in combination with ultrasound. The assessment includes VAS score, CSI score, SQS score, SF-36 score, technical parameters and postoperative complications.

#### Statistical analysis

Statistical analysis was performed using the SPSS analysis software (version 27.0 SPSS).

Mean ± standard deviation values are used to represent numeric variables, while frequencies and percentages are utilized to describe categorical variables. Different statistical tests such as Mann–Whitney U test, independent *t*-test, chi-squared test, and Fisher’s exact test were employed to compare groups. A *p* value less than 0.05 was deemed to be statistically significant.

## Results

### Patient demographics

In the study, 212 patients with perineal HZ were enrolled, while 19 patients were excluded for various reasons: one patient had lumbar vertebral tumor, eight patients had incomplete information, four patients had pain lasting more than 3 months, and six patients were over the age of 85. A total of 193 patients were ultimately included in the study (Figure 1).

The included patients were divided into two groups: DSA group (*n* = 87) and ultrasound + DSA group (*n* = 106). A total of 16 patients were lost to follow-up (Figure 1). There was no statistically significant variance between the baseline and post-treatment in terms of demographic data and patient characteristics (Table 1). It is important to note that 4 patients (2.1%) had fecal incontinence, and 3 (1.6%) had urinary incontinence at the time of admission.

**Table 1:** Demographic and clinical characteristics data.

Variable	Baseline (*n* = 193)	Post-treatment (*n* = 177)	*p*
Age (mean ± SD, y)	66.9 ± 9.5	66.8 ± 9.5	0.981
Sex (M/F)	81/112	73/104	0.887
Specific comorbidities* (*n*, %)			
Hypertension	75 (38.9%)	72 (40.7%)	0.721
Diabetes	34 (17.6%)	32 (18.1%)	0.908
Cardiac disease	31 (16.1%)	27 (15.3%)	0.831
Kidney disease	12 (6.2%)	12 (6.8%)	0.826
Pulmonary disease	38 (19.7%)	34 (19.2%)	0.907
Liver disease	8 (4.1%)	7 (4.0%)	0.926
HZ Location (*n*, %)			
S2	90 (46.6%)	83 (46.9%)	0.960
S3	99 (51.3%)	94 (53.1%)	0.727
S4	4 (2.1%)	0 (0%)	0.124
Duration of pain (mean ± SD, d)	16.7 ± 15.8	16.4 ± 15.7	0.792
Anatomical site of zoster (*n*, %)			0.879
Left	104 (56.8%)	102 (57.6%)	
Right	79 (43.2%)	75 (42.4%)	
Antiviral record within 7 days of zoster (*n*, %)	180 (93.3%)	169 (95.5%)	0.357
Fecal incontinence (*n*, %)	4 (2.1%)	1 (0.6%)	0.374
Urinary incontinence (*n*, %)	3 (1.6%)	0 (0%)	0.249

*Combination of multiple underlying diseases in one patient.

### Pain evaluation

**Figure 3:**
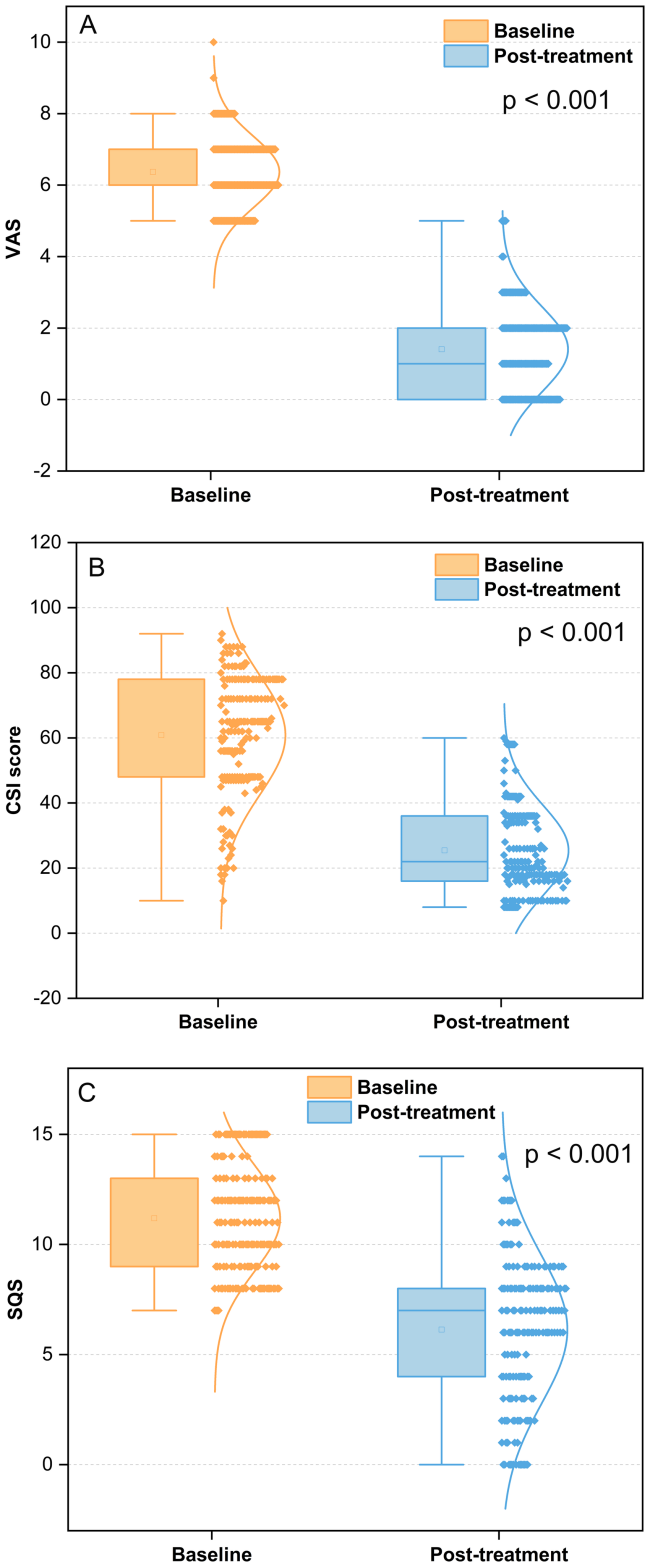
Outcomes of VAS, CSI, and SQS at baseline and post-treatment. (A) Pain intensity at post-treatment vs. the baseline, *p* < 0.001; (B) central sensitization score at post-treatment vs. the baseline, *p* < 0.001; (C) sleep problems score at post-treatment vs. the baseline, *p* < 0.001; VAS, Visual analog scale; CSI, Central sensitization inventory; SQS, Sleep quality scores.

**Figure 4:**
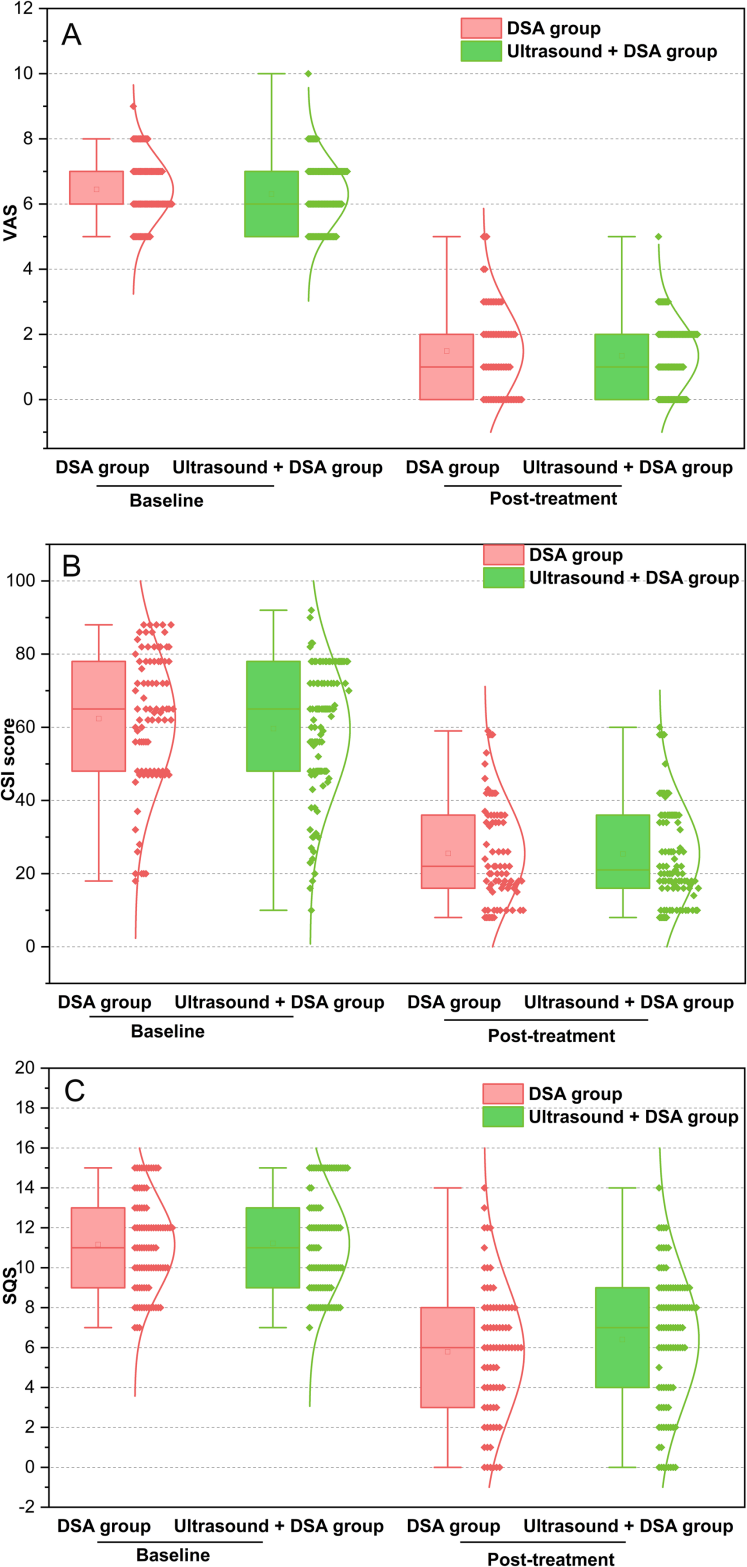
Outcomes of VAS, CSI, and SQS in the DSA group and ultrasound + DSA group at baseline and post-treatment. A: pain intensity in the two groups at post-treatment vs. the baseline, *p* > 0.05; (B) central sensitization score in the two groups at post-treatment vs. the baseline, *p* > 0.05; (C) sleep problems score in the two groups at post-treatment vs. the baseline, *p* > 0.05; VAS, Visual analog scale; CSI, Central sensitization inventory; SQS, Sleep quality scores.

The mean pain score was statistically significantly lower after treatment compared to baseline (6.4 ± 1.0 vs. 1.4 ± 1.2, *p* < 0.001) (Figure 3A). At baseline, the mean pain score in the DSA group was 6.4 mildly higher than in the ultrasound + DSA group (6.3), which was not statistically significant (*p* = 0.331). Likewise, there were no significant differences in pain scores between the two groups at the end of the follow-up period (1.5 ± 1.3 vs. 1.3 ± 1.1, *p* = 0.705) (Figure 4A).

### Central sensitization evaluation

Perineal HZ patients displayed marked improvements in CSI scores from baseline to post-treatment (*p* < 0.001) (Figure 3B). We additionally analyzed the CSI score curve for both the DSA group and the ultrasound + DSA group. Although patients in the DSA group showed higher CSI score than the ultrasound + DSA group at baseline, it was not statistically significant (62.4 ± 18.4 v s 59.6 ± 18.1, *p* = 0.325). Nevertheless, there was no significant change in CSI scores for either group following the treatment (25.5 ± 14.0 vs. 25.4 ± 13.7, *p* = 0.976) (Figure 4B).

### Sleep quality evaluation

Perineal HZ patients showed significant reduction in SQS score between baseline and post-treatment (11.2 ± 2.4 vs. 6.1 ± 3.4, *p* < 0.001) (Figure 3C).

We compared the SQS scores between the DSA group and the ultrasound + DSA group. At baseline, the SQS scores in the DSA group were similar to those in the ultrasound + DSA group (11.2 ± 2.4 vs. 11.2 ± 2.5, *p* > 0.05). At post-treatment, although the SQS score in the DSA group was lower than the SQS score in the ultrasound + DSA group, it was not statistically significant (5.8 ± 3.3 vs. 6.4 ± 3.2, *p* > 0.05) (Figure 4C).

### Quality of life evaluation

The SF-36 scale assesses eight dimensions of health-related quality of life, including physical function (PF), role physical (RP), bodily pain (BP), general health (GH), vitality (V), social function (SF), role of emotion (RE), and mental health (MH) (18). The results demonstrated that there were significant improvements in eight dimensions of SF-36 after PRF treatment compared to the initial baseline measurement (*p* < 0.001) (Table 2). However, there was no significant difference in between the DSA group and ultrasound + DSA group at baseline and post-treatment in respect to the quality of life (*p* > 0.05) (Table 3).

**Table 2:** Results of quality-of-life changes between baseline and post-treatment.

Variable	Baseline (*n* = 193)	Post-treatment (*n* = 177)	*p*
PF	35.8 ± 17.4	62.3 ± 19.0	<0.001
RP	17.7 ± 19.2	58.2 ± 22.7	<0.001
BP	39.1 ± 13.7	61.2 ± 18.1	<0.001
GH	25.5 ± 19.1	54.5 ± 18.5	<0.001
V	28.8 ± 15.2	61.5 ± 14.3	<0.001
SF	34.0 ± 21.3	69.1 ± 16.3	<0.001
RE	27.1 ± 26.6	52.0 ± 25.3	<0.001
MH	27.9 ± 21.2	75.3 ± 17.6	<0.001

PF, Physical function; RP, Role physical; BP, Bodily pain; GH, General health; V, Vitality; SF, Social function; RE, Role of emotion; MH, Mental health.

**Table 3:** Results of quality-of-life changes from baseline to follow-up between the DSA group and ultrasound + DSA group.

Variable	Baseline		*p*	Post-treatment		*p*
	DSA group (*n* = 87)	Ultrasound + DSA group (*n* = 106)		DSA group (*n* = 78)	Ultrasound + DSA group (*n* = 99)	
Age (mean ± SD, y)	66.7 ± 8.5	67.0 ± 10.3	0.428	66.7 ± 8.3	66.9 ± 10.4	0.512
Sex (M/F)	38/49	43/63	0.663	33/45	40/59	0.798
PF	34.0 ± 18.7	37.3 ± 16.1	0.182	64.6 ± 16.7	60.5 ± 20.5	0.165
RP	16.0 ± 18.6	19.1 ± 19.7	0.364	62.2 ± 19.2	55.1 ± 24.7	0.094
BP	40.3 ± 13.8	38.1 ± 13.5	0.297	62.6 ± 16.6	60.1 ± 19.2	0.542
GH	28.0 ± 21.3	23.4 ± 16.9	0.453	56.2 ± 18.6	53.1 ± 18.3	0.307
V	27.9 ± 16.9	29.5 ± 13.7	0.176	65.6 ± 12.5	58.3 ± 14.9	0.158
SF	32.2 ± 23.1	35.5 ± 19.7	0.725	69.4 ± 16.0	68.9 ± 16.7	0.694
RE	26.0 ± 25.2	28.0 ± 27.8	0.705	59.0 ± 22.9	46.5 ± 25.7	0.224
MH	31.3 ± 22.7	25.2 ± 19.5	0.264	77.0 ± 15.4	74.0 ± 19.1	0.388

PF, Physical function; RP, Role physical; BP, Bodily pain; GH, General health; V, Vitality; SF, Social function; RE, Role of emotion; MH, Mental health.

### Technical parameters and adverse events

The time it took from the start of the puncture to the successful puncture was significantly shorter in the ultrasound + DSA group compared to the DSA group (41.2 ± 21.2 vs. 48.1 ± 20.3, *p* = 0.035). Patients rated their overall satisfaction with the procedure on a scale from 0 (not satisfied at all) to 100 (very satisfied). Patient satisfaction was defined as a score of 90 or higher. The results showed that the ultrasound + DSA group had a higher percentage of satisfaction with the procedure than the DSA group (90/99, 90.9% vs. 62/78, 79.5%, *p* = 0.030) (Table 4).

**Table 4:** Technical parameters and postoperative complications.

Variable	DSA group (*n* = 78)	Ultrasound + DSA group (*n* = 99)	*p*
Puncture time taken* (min)	48.1 ± 20.3	41.2 ± 21.2	0.035
Patient satisfaction	62 (79.5%)	90 (90.9%)	0.030
Postoperative complications			
Puncture site hematoma	10 (12.8%)	4 (4.0%)	0.032
Puncture site infection	2 (2.6%)	1 (1.0%)	0.584
Local skin numbness	4 (5.1%)	3 (3.0%)	0.701
Weak or numbness of lower limbs	2 (2.6%)	1 (1.0%)	0.584

*Time from start of puncture to successful puncture; &: Patients rated their overall satisfaction with the procedure on a scale from 0 (not satisfied at all) to 100 (very satisfied). Patient satisfaction was defined as a score of 90 or higher.

A total of 27 postoperative complications occurred, such as 14 cases of hematoma at the puncture site, 3 cases of infection at the puncture site, 7 cases of local skin numbness, and 3 cases of lower limb weakness or numbness. It was evident that there was a significantly higher hematoma at the puncture site in the DSA group (10/78, 12.8%) compared to the ultrasound + DSA group (4/99, 4.0%), demonstrating statistical significance (*p* = 0.032). There was no statistically significant difference in other complications between the two groups (Table 4).

## Discussion

This study presents the first report on the treatment of HZ-related pain in the perineal area using PRF. The findings indicate that DSA-guided PRF effectively alleviates HZ-related pain in the perineal region, reduces central sensitization, enhances sleep quality, and improves overall quality of life. Notably, the combination of PRF treatment under ultrasound guidance with DSA guidance can reduce puncture time and increase patient satisfaction with the procedure.

Pulsed radiofrequency is a minimally invasive interventional procedure. It is one of the most commonly used treatments for chronic pain, such as herpes zoster (27), radicular pain (28, 29), trigeminal neuralgia (30), occipital neuralgia (31), and joint pain (32, 33). According to the research, PRF lowers pain perceptions by sending electrical impulses of a precise frequency and voltage to neurons or tissues, hence altering nerve conduction and neuron excitability. Pulsed radiofrequency causes less thermal damage to tissues than standard radiofrequency treatments, lowering the risk of nerve damage and other problems. This is because PRF’s electrical energy has no substantial temperature impact on tissues (34, 35). Hagiwara et al. (36) found that PRF analgesic effects included an increase in noradrenergic and 5-hydroxytryptaminergic downstream pain inhibitory pathways. Vallejo et al. (34) discovered that PRF treatment reduces pain by using electromagnetic energy to affect the behavioral and molecular impacts of hypersensitive reactions caused by peripheral nerve damage. In addition, multiple studies have demonstrated that RF slows and reverses myelin degradation (37, 38). Although the underlying mechanisms have not been elucidated, the PRF technique is clinically effective and has gained acceptance among clinical physicians.

Rash and pain are the main symptoms of herpes in the perineal area. A small percentage of patients experience urinary and fecal incontinence. Pain is one of the most significant elements influencing patients’ quality of life and sleep. As a result, management of shingles pain is one of the key indicators of the effectiveness of treatment for HZ in the perineal area. In our study, PRF can relieve HZ-related pain in the perineal area. The results showed that the mean pain score was statistically significantly lower after PRF treatment compared to baseline (*p* < 0.001). In addition, we were surprised to discover that PRF could improve the patient’s urinary and fecal incontinence. In the study, seven patients had urine and fecal incontinence. After PRF treatment, 6 patients improved their fecal or urine function, while only 1 patient with fecal incontinence exhibited no improvement in symptoms, which may be related to the long duration of pain. Therefore, early PRF treatment not only alleviates HZ-related pain, but also helps to improve urinary and fecal incontinence.

Central sensitization is considered to be an underlying pathophysiological mechanism in a group of chronic pain conditions and may be an important factor in the promotion of chronic pain (39). Evidence for central sensitization in many chronic pain-related diseases, such as osteoarthritis (40), headache (41), chronic neck pain (42), complex regional pain syndrome (43). The effect of central sensitization on neuropathic pain has been less well studied. Campbell et al. (44) first demonstrated the possible role of central sensitization in neuropathic pain. A study by Liu and colleagues has identified that the CXCL12/CXCR4 signaling pathway promoted the development and maintenance of neuropathic pain through central sensitization mechanisms (45). In the study, we have reported for the first time that PRF can alleviate central sensitization caused by HZ-related pain. The results confirm that perineal HZ patients displayed marked improvements in CSI scores from baseline to post-treatment (*p* < 0.001). What puzzles us, however, is the pathway by which PRF reduces central sensitization, and the underlying mechanisms need to be further investigated.

Quality of life evaluation is commonly used in clinical pain medicine, mainly due to its ability to reflect the subjective and multifaceted character of chronic pain. There are various tools for assessing quality of life in patients with HZ, including the SF-12 (12-item Short Form Survey) (46), EQ-5D (EuroQol-5 Dimensions) (47), activities of daily living questionnaires (19), SF-36 (Short Form Health Survey 36) (48), EORTC QLQ-30 (The European Organization for Research and Treatment of Cancer QOL Core Questionnaire 30) (49). However, there is no consensus on standard health-related quality of life measurement. This study was conducted to assess the quality of life of the patients through SF-36 questionnaire. Previous study has confirmed the reliability of the SF-36 and for construct validity in terms of distinguishing between groups with expected health differences (50). The SF-36 is a generic instrument containing eight individual subscales divided into physical and psychological domains: physical function (PF), role physical (RP), bodily pain (BP), general health (GH), vitality (V), social function (SF), role of emotion (RE), and mental health (MH) (26). The results of our study showed that PRF can improve quality of life in patients with perineal zoster-associated pain. Further, the study has found that sleep quality is associated with quality of life. Tsuru et al. (51) have showed that poor sleep quality was linked to a poor health-related quality of life in Parkinson’s disease. The results from nationwide Singapore Mental Health Study indicated that poor Pittsburgh Sleep Quality Index (PSQI) score was significantly associated with low physical component summary (PCS) and low mental component summary (MCS) (52). We found that patients not only had an improved quality of life after PRF treatment, but also a simultaneous improvement in sleep quality. Therefore, timely and appropriate PRF treatment for HZ in the perineal area is beneficial.

Recently, ultrasound technique has shown unique advantages in clinical pain management. Compared with traditional imaging modalities such as X-ray, computed tomography (CT), and magnetic resonance imaging (MRI), ultrasound guidance has the benefits of no ionizing radiation, real-time imaging, portability, and low cost. Precise positioning of the puncture needle around the sacral nerve is a key step in the success of PRF. Finding the posterior sacral foramen under DSA guidance is the first step in the puncture procedure. Discovering the posterior sacral foramen and puncturing it to reach the anterior foramen is a difficult task due to the anatomical characteristics and variability of sacral structures. Repeated multiple punctures can lead to long procedure times, local hematoma or neurovascular injury. With the development of technology, ultrasound is a good solution to this problem. The posterior sacral foramen can be found quickly and precisely under ultrasound guidance. Due to the limited view of the puncture plane under ultrasound guidance, the puncture channel cannot be fully visualized and the depth of the puncture needle cannot be displayed. Ultrasound-guided puncture may be too shallow to achieve therapeutic effect or too deep to cause pelvic tissue damage. In this study, PRF treatment was performed under ultrasound guidance in combination with DSA, with shorter puncture time and fewer complications than PRF treatment under DSA guidance alone. Therefore, we suggest that patients with HZ in the perineal area should combine ultrasound and DSA when undergoing PRF therapy.

This study has some limitations. First, the sample size of this study was small. On the one hand, the prevalence of HZ in the perineal area is lower than that of HZ in the thoracolumbar region. On the other hand, some patients may refuse to seek medical attention because the pain was located in a private area. Second, we did not discuss the relationship between pain and quality of life. It is generally accepted that pain can affect quality of life, but it is not clear whether pain improvement ameliorates quality of life. An observational prospective study has found that pain changes were not related to changes of SF-36 (53). Therefore, our next step is to focus on the relationship between pain and quality of life in HZ in the perineal area.

## Conclusion

PRF can reduce pain from HZ in the perineal region, alleviate central sensitization, enhance sleep quality, and improve overall quality of life. When PRF is performed under ultrasound guidance combined with DSA, it shortens the puncture time and reduces the risk of hematoma formation at the puncture site, making it the recommended method for clinical use.

## Data Availability

The raw data supporting the conclusions of this article will be made available by the authors, without undue reservation.
